# Successful pancreatectomy after conversion-intended chemotherapy using gemcitabine and nab-paclitaxel for unresectable adenosquamous carcinoma of the pancreas: a case report

**DOI:** 10.1186/s40792-024-01989-5

**Published:** 2024-08-16

**Authors:** Kenichi Nakamura, Mitsuru Nakagawa, Mizuki Ariga, Takahiko Higashiguchi, Yuko Chikaishi, Kazuhiro Matsuo, Aki Nishijima, Tomoyoshi Endo, Kenji Kikuchi, Koji Morohara, Hidetoshi Katsuno, Yoshihiko Tachi, Ichiro Uyama, Koichi Suda, Zenichi Morise

**Affiliations:** 1 Department of Surgery, Fujita Health University Okazaki Medical Center, 1 Azakotanda, Harisaki, Okazaki, Aichi 444-0827 Japan; 2 Department of Surgery, Fujita Health University, 1-98 Dengakugakubo, Kutsukake, Toyoake, Aichi 470-1192 Japan; 3 Department of Pathology, Fujita Health University Okazaki Medical Center, 1 Azakotanda, Harisaki, Okazaki, Aichi 444-0827 Japan; 4 Department of Gastroenterology, Fujita Health University Okazaki Medical Center, 1 Azakotanda, Harisaki, Okazaki,, Aichi 444-0827 Japan; 5 Department of Advanced Robotic and Endoscopic Surgery, Fujita Health University, 1-98 Dengakugakubo, Kutsukake, Toyoake, Aichi 470-1192 Japan

**Keywords:** Carcinoma, Adenosquamous, Chemotherapy, Neoadjuvant therapies, Pancreatic cancer, Chemotherapy

## Abstract

**Background:**

Adenosquamous carcinoma of the pancreas (ASCP) accounts for only 1–4% of all pancreatic exocrine cancers and has a particularly poor prognosis. The efficacy of chemotherapy for ASCP remains unknown because of the small number of cases, and few studies have evaluated conversion-intended chemotherapy.

**Case presentation:**

A 76-year-old woman was referred to our hospital because of epigastric pain and nausea. A preoperative contrast-enhanced multidetector row computed tomography (MDCT) scan revealed a 17 × 17 mm low-density tumor with an ill-defined margin at the arterial phase in the pancreatic head. The tumor involved the common hepatic artery, left hepatic artery bifurcated from the common hepatic artery, and gastroduodenal artery, and was in contact with the portal vein. Fluorodeoxyglucose-positron emission tomography (FDG-PET) showed an uptake in the pancreatic head but no evidence of distant metastasis. The tumor was diagnosed as an adenocarcinoma of the pancreatic head and staged unresectable because the common and left hepatic arteries were involved. Hence, the patient underwent seven courses of conversion-intended chemotherapy using gemcitabine and nab-paclitaxel for pancreatic ductal adenocarcinoma over 7 months. After chemotherapy, the tumor shrank to 10 × 10 mm on contrast-enhanced MDCT. Consequently, the boundary between the tumor and major vessels of the common and left hepatic arteries and the portal vein became clear, and the involvement of the arteries with the tumor was evaluated to be released. The contact of the tumor to the portal vein also reduced to less than half the circumference of the portal vein. FDG-PET showed decreased accumulation in the tumor. Hence, the tumor was judged resectable, and pancreaticoduodenectomy was performed. The tumor and major blood vessels were easily dissected and R0 resection was achieved. The patient experienced no major complications and was discharged on postoperative day 28. The tumor was revealed as ASCP via pathological examination. The patient is alive and recurrence-free seven months after surgery. This is the first report of successful R0 resection for an initially unresectable ASCP following conversion-intended chemotherapy using gemcitabine and nab-paclitaxel regimen.

**Conclusions:**

Conversion-intended chemotherapy using gemcitabine and nab-paclitaxel regimen may be effective for ASCP.

## Background

Adenosquamous carcinoma of the pancreas (ASCP) accounts for only 1–4% of all pancreatic exocrine cancers and has a particularly poor prognosis [1]. The median overall survival after resection is reported to be 6.8 months while the 3-year overall survival is 14.0% [2]. Boyd et al. reported that the 2-year survival for patients with ASCP after resection is worse than that for pancreatic ductal adenocarcinoma (PDAC) (29% vs. 36%; P < 0.0001) [3]. Since ASCP is a rare tumor, no tailored treatment guidelines have been established [4], and therapy often follows treatment guidelines for PDAC [5]. Thus, ASCP treatment may involve a combination of surgery, chemotherapy, and radiation [5]. However, a consensus on chemotherapy for ASCP has not been established, and few studies have reported on conversion-intended chemotherapy. Herein, we present a successful R0-pancreatectomy for initially unresectable ASCP after conversion-intended chemotherapy using gemcitabine and nab-paclitaxel regimen.

## Case presentation

**Fig. 1:**
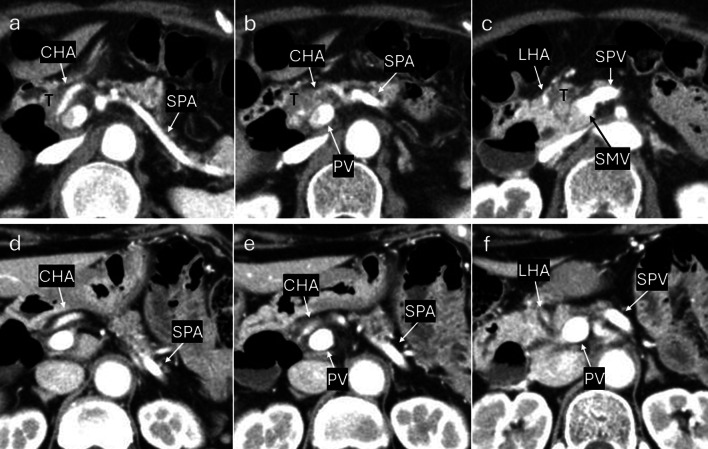
Multidetector row computed tomography (MDCT) scan findings of a pancreatic adenosquamous carcinoma. **a**–**c** Contrast-enhanced MDCT scan findings before conversion-intended chemotherapy. The tumor had invaded the common and left hepatic arteries and contacted more than half the circumference of the portal vein. **d**–**f** Contrast-enhanced MDCT scan findings after conversion-intended chemotherapy. The tumor in the pancreatic head shrank to release the involvement of the common hepatic and left hepatic artery. The contact with the tumor to the portal vein became less than half the circumference of this vein. T, tumor; CHA, common hepatic artery; SPA, splenic artery; PV, portal vein; LHA, left hepatic artery; SPV, splenic vein; and SMV, superior mesenteric vein

**Fig. 2:**
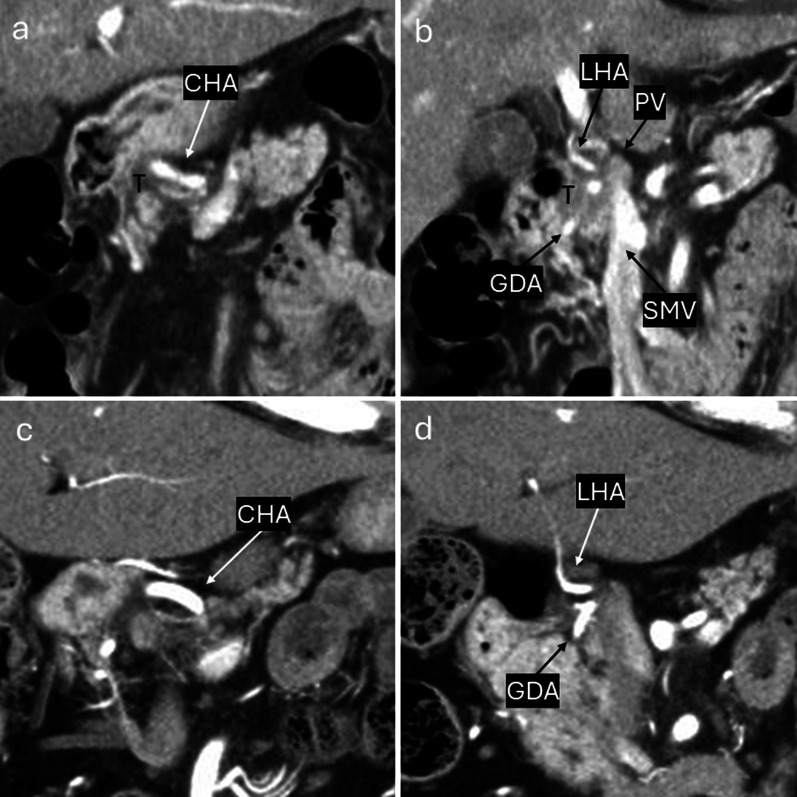
Multidetector row computed tomography (MDCT) scans of a pancreatic adenosquamous carcinoma. **a**, **b** Contrast-enhanced MDCT scans before conversion-intended chemotherapy. The tumor had invaded the common hepatic, left hepatic, and gastroduodenal arteries and contacted the superior mesenteric and portal veins. **c**, **d** Contrast-enhanced MDCT scans after conversion-intended chemotherapy. The margin of the common and left hepatic arteries became distinct, and clear fat planes around these arteries were observed. GDA, gastroduodenal artery

**Fig. 3:**
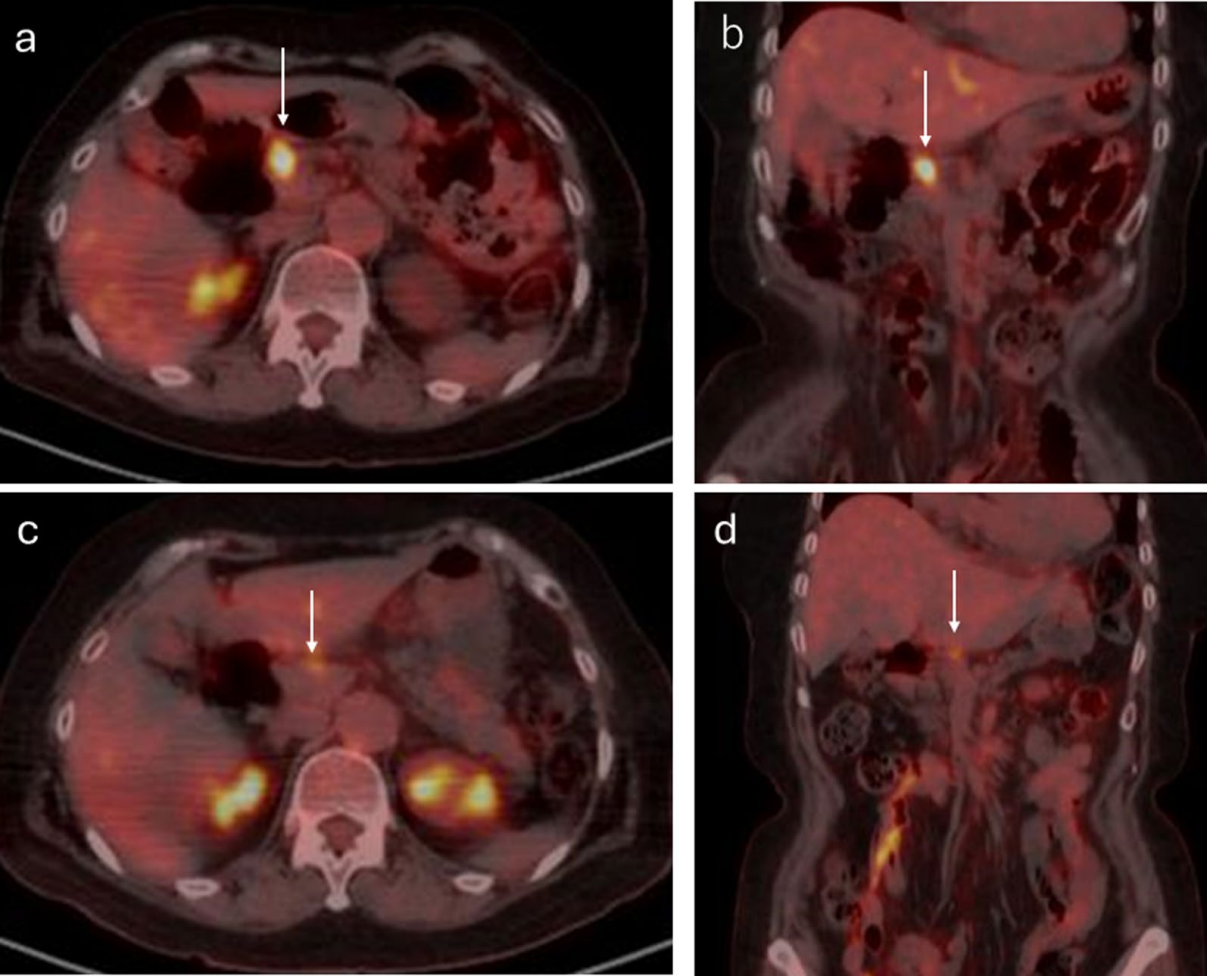
Fluorodeoxyglucose-positron emission tomography (FDG-PET) findings. **a**, **b** Before neoadjuvant chemotherapy, FDG-PET showed a standardized uptake maximum value of 6.6 in the pancreatic head. **c**, **d** After chemotherapy, FDG-PET showed a decreased accumulation with a standardized uptake maximum of 3.6 in the tumor

**Fig. 4:**
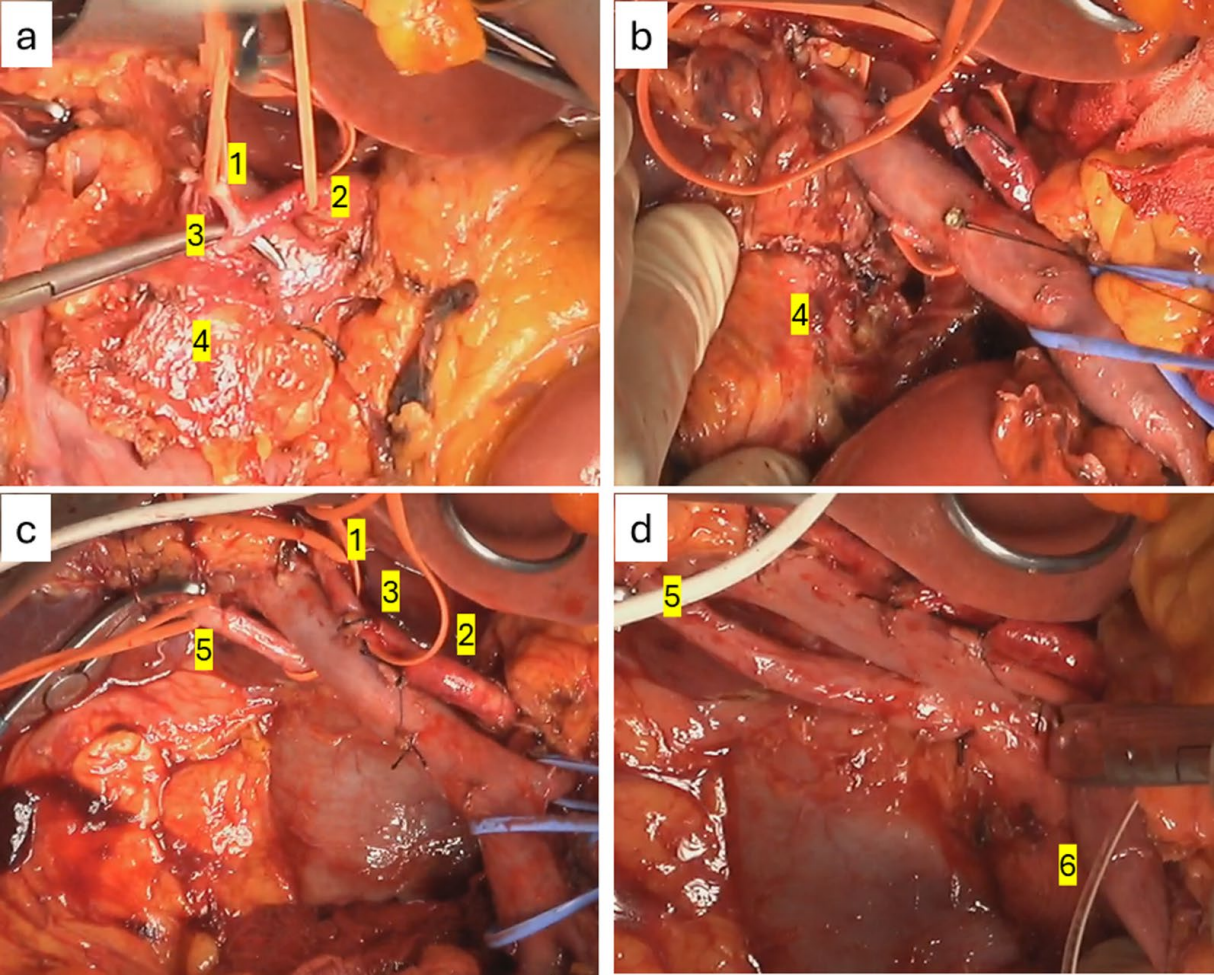
Intraoperative findings. **a** The tumor did not invade the common or left hepatic arteries and was easily dissected. **b** The tumor in the pancreatic head did not invade the portal vein and could be dissected without resection of the portal vein. **c** The right hepatic artery was bifurcated from the celiac artery and showed no tumor invasion. **d** The tumor did not invade the superior mesenteric artery. Finally, R0 resection was performed. 1. Left hepatic artery, 2. common hepatic artery, 3. gastroduodenal artery, 4. pancreas head, 5. right hepatic artery, and 6. superior mesenteric artery

**Fig. 5:**
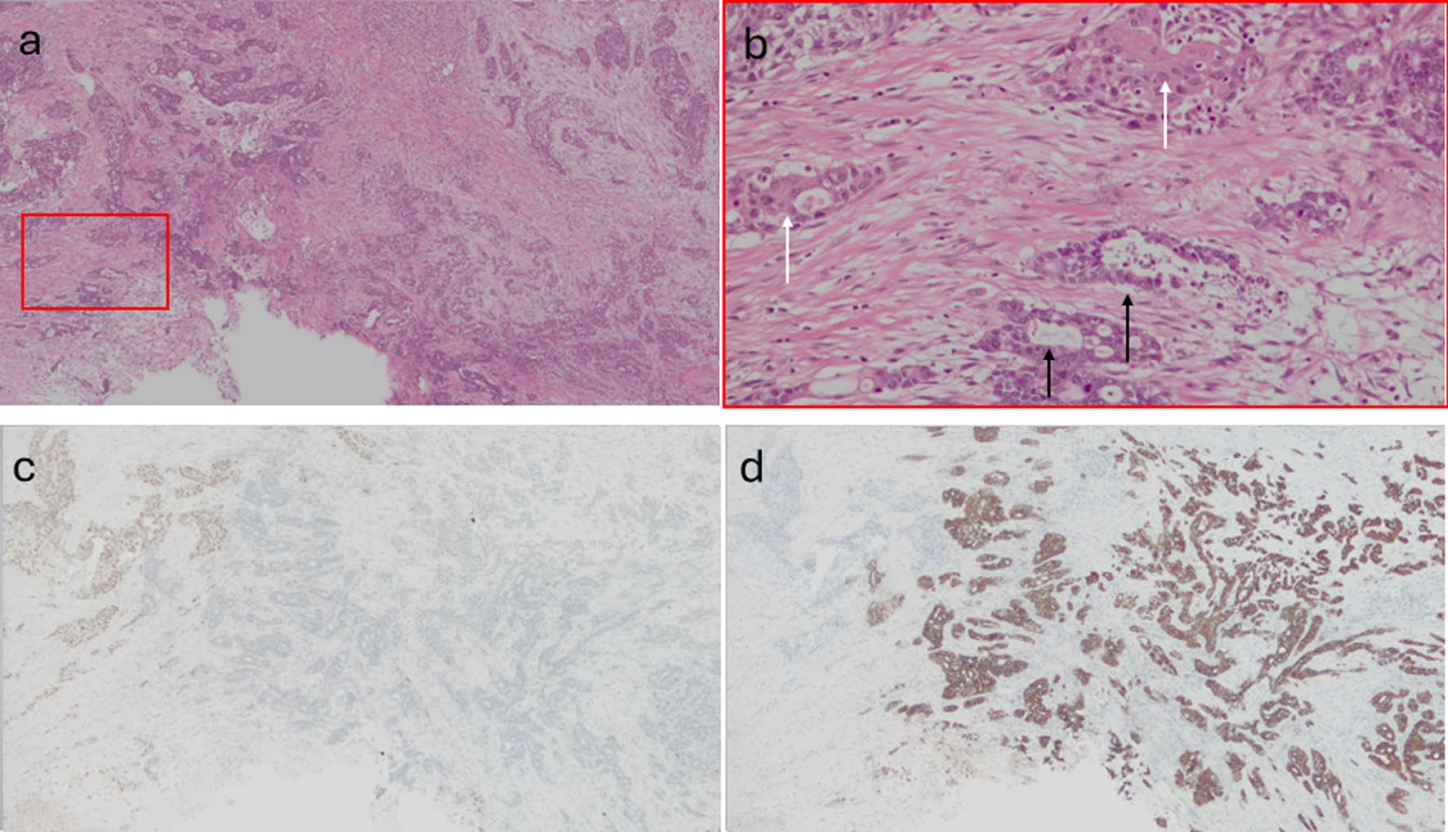
Histological and immunohistochemical results of adenosquamous carcinoma of the pancreas. Hematoxylin and eosin staining (**a**, 20 ×) and (**b**, 200 ×) revealed the adenocarcinoma component forming glandular structures and the squamous cell carcinoma component displaying nests, which were partially intermingled with continuity. Black arrow: adenocarcinoma; white arrow: squamous cell carcinoma. **c** Immunohistochemical p40 staining is positive for squamous cell carcinoma and negative for adenocarcinoma. **d** CK7 staining is positive for adenocarcinoma and negative for squamous cell carcinoma

A 76-year-old woman was referred to our hospital because of epigastric pain and nausea. The medical history included myomectomy of the uterus and osteoporosis, but no malignancies, including squamous cell carcinoma. A preoperative multidetector row computed tomography (MDCT) scan revealed a 17 × 17 mm low-density tumor with an ill-defined margin at the arterial phase in the pancreatic head (Figs. 1a–c and 2a, b). The left hepatic artery and gastroduodenal artery were bifurcated from the common hepatic artery, and the right hepatic artery was directly bifurcated from the celiac artery. The tumor involved the common hepatic, left hepatic, and gastroduodenal arteries and was in contact with the portal vein (Figs. 1a–c and 2a, b). Abdominal ultrasonography revealed a hypoechoic tumor of the same size as shown by MDCT in the pancreatic head. Fluorodeoxyglucose-positron emission tomography (FDG-PET) showed an uptake (standardized uptake value max 6.6) in the pancreatic head but no evidence of distant metastasis (Fig. 3a, b). Blood tests demonstrated an elevated level of CA19-9 (CA19-9 103.4 U/mL, CEA 2.0 ng/mL, DUPAN2 < 25 U/mL, and SCC antigen and CYFRA unmeasured). The tumor was diagnosed as a locally advanced adenocarcinoma of the pancreatic head and was unresectable because of the involvement of the common and left hepatic arteries. Endoscopic ultrasonography-fine needle aspiration (EUS-FNA) was not performed because of the concerns about needle tract seeding and the potential for a delay in treatment resulting from the time required for pathological diagnosis or complications [6]. Thus, the patient initially underwent three courses of chemotherapy using gemcitabine and nab-paclitaxel for PDAC to downstage the tumor over three months. Consequently, an MDCT scan showed a partial response, although this was insufficient to be diagnosed as resectable. Given the efficacy of the chemotherapy, the same regimen was continued in anticipation of further downstaging while also monitoring for the absence of distant metastases. After seven courses of chemotherapy over seven months, the tumor markedly shrank to 10 × 10 mm in size on contrast-enhanced MDCT. Consequently, the involvement of the common and left hepatic arteries of the tumor was released and clear fat planes around them were observed (Figs. 1d–f and 2c, d) [7]. Furthermore, contact with the tumor to the portal vein reduced to less than half the circumference of the portal vein. FDG-PET showed decreased accumulation (standardized uptake value max 3.6) in the pancreatic head (Fig. 3c, d) and no other positive lesions. These findings suggested that the tumor became resectable with pancreaticoduodenectomy after the treatment [7, 8]. Blood tests showed a significant decrease in CA19-9 levels (29.2 U/mL). Consequently, pancreaticoduodenectomy was performed for this downstaged cancer with resectability. Although lavage cytology was not performed, open intra-abdominal inspection did not reveal serosal invasion of the tumor or peritoneal nodules, leading to a diagnosis of an absence of peritoneal dissemination. During the operation, while excising the nerves around the arteries (the nerves of the SMA were resected semicircumferentially), resection was performed along the layer exposing the adventitia of the blood vessels because they were initially invaded by the tumor. No findings, such as sclerosis of the nerves or adhesion of the nerves to surrounding tissues or the adventitia of the vessels, were suggestive of perivascular nerve invasion of the major arteries, and the dissection between the adventitia of the vessels and the nerves was performed easily, although a frozen section diagnosis of the nerves around the major arteries was not performed. Consequently, the tumor and major blood vessels, including the common and left hepatic arteries, superior mesenteric artery, and portal vein were easily dissected. Finally, R0 resection was achieved (Fig. 4). Pathological examination revealed that the tumor was an ASCP and was characterized by an admixture of distinct components of both adenocarcinoma and squamous cell carcinoma, with partial continuity between them (Fig. 5). The squamous cell carcinoma component represented 30% of the tumor. The tumor was resected with negative tumor margins, and measured 24 × 18 mm, with no lymph node metastasis detected (ypT2N0M0, yp Stage IB) [9]. No evidence was present of nerve invasion around the common and left hepatic arteries and the superior mesenteric artery. The residual tumor was viable, but fibrosis was present around the tumor without extrapancreatic invasion. The grading of histological response to preoperative therapy was Grade IIa in the Evans classification [10].

The patient experienced no major complications and was discharged on postoperative day 28. The patient was administered S-1 orally as an adjuvant chemotherapy. At seven months postoperatively, the patient is alive and free of any radiological imaging evidence of recurrence or elevation of tumor markers (CA19-9, DUPAN2, CEA, SCC antigen, and CYFRA).

## Discussion

Clinical manifestations of ASCP are similar to those of PDAC and include abdominal pain, jaundice, and weight loss [11]. Accurate preoperative diagnosis of ASCP is difficult because imaging studies cannot reveal the specific features that differentiate them from PDAC, although several useful indicators have been reported, including an infiltrating round globular mass with extensive central necrosis [2, 12, 13]. Presently, the preoperative diagnosis of ASCP relies on histopathological examination via FNA or endoscopic retrograde pancreatic juice aspiration, but reports of these are rare [2, 14, 15]. According to World Health Organization criteria, the percentage of squamous cell carcinoma must be at least 30% for ASCP [16]. ASCP has historically been considered clinically more aggressive with a poorer prognosis than PDAC [3]. The squamous component is considered to worsen the prognosis because malignant squamous cells grow at twice the rate of adenocarcinoma cells and are more likely to demonstrate vascular invasion [17, 18]. Tumors in patients with ASCP tended to be larger than PDAC tumors, with a 50% chance of metastatic disease at initial presentation [19]. Features such as central necrosis and hypervascularity are often seen in patients with ASCP, along with high tumor grade [20, 21]. However, Boyd et al. reported that surgical resection still offers the best chance of cure and long-term survival [3]. However, unlike PDAC, no standard adjuvant therapy has been established for ASCP [1, 19, 22–24]. Hue et al. noted the importance of multimodality therapy combining chemotherapy and pancreatectomy in patients with nonmetastatic ASCP in a study based on the National Cancer Database (U.S.A, 2010–2016) [25]. Of the 838 patients, 64.7% underwent pancreatectomy, and of these, 60.5% received adjuvant chemotherapy, 14.8% (80 patients) received neoadjuvant chemotherapy, and 24.7% received surgery only. The median survival of patients who received chemotherapy alone was comparable with those who received pancreatectomy alone (9.2 vs. 7.2 months, *P* = 0.504). Overall survival was improved when patients received both chemotherapy and pancreatectomy (neoadjuvant = 19.6 months, hazard ratio = 0.58; adjuvant = 19.4 months, hazard ratio = 0.64) compared with pancreatectomy alone. However, the details of chemotherapy, including regimen and number of cycles were not analyzed since the National Cancer Database did not record this. Fang et al. reported in a retrospective cohort study for ASCP that adjuvant chemoradiotherapy significantly improved overall survival compared with adjuvant single therapy (chemo or radiotherapy) (44 patients, 23 months vs. 91 patients, 13 months, *P* = 0.012) [26].

In the present case, the patient was initially diagnosed with unresectable PDAC because of major vascular involvements, and conversion-intended chemotherapy was selected, with gemcitabine and nab-paclitaxel regimen used as per PDAC treatment. To achieve downstaging for resectability and monitor for the absence of distant metastases, seven courses of chemotherapy were administered over seven months. Consequently, the tumor was downsized, and vascular involvements of the tumor were solved to enable R0 resection to be achieved. The first choice of chemotherapy for PDAC is a four-drug combination of leucovorin, 5-fluorouracil, irinotecan, and oxaliplatin (FOLFIRINOX) or a two-drug combination of gemcitabine and nab-paclitaxel [5]. In chemotherapy for ASCP, Paredes et al. reported a case of unresectable ASCP where combination therapy with gemcitabine, nab-paclitaxel, and pembrolizumab, as a second-line regimen after progression of disease with FOLFIRINOX, produced a dramatic response in tumor size and biomarkers and ultimately enabled maintenance therapy with pembrolizumab [5]. Regarding adjuvant chemotherapy for ASCP, most case reports in the literature have used 5-fluorouracil-based therapy. The role of gemcitabine or more potent regimens such as FOLFIRINOX or nab-paclitaxel/gemcitabine has rarely been examined [21, 27]. In a cohort study of 62 (1.1%) patients undergoing pancreatectomy out of 5627 patients with ASCP, Wild et al. suggested that overall survival may be improved by the addition of platinum agents to a 5-FU or gemcitabine-based chemotherapy regimen in adjuvant chemotherapy [28]. However, few studies have evaluated the use of preoperative chemotherapy including neoadjuvant and conversion-intended chemotherapy, and its effects are poorly understood [29]. To the best of our knowledge, our study is only one of three cases where tumor resection was successfully performed after chemotherapy for initially unresectable ASCP [30, 31]. Tanaka et al. reported that an unresectable ASCP partially responded to chemotherapy that combined interferon-α, tumor necrosis factor-α, and 5-fluorouracil and finally received a pancreaticoduodenectomy [30]. However, the tumor rapidly recurred, and the patient survived for only seven months after the operation; therefore, preoperative chemotherapy might not have contributed to prolonged patient survival. Paramythiotis et al. performed chemotherapy using FOLFIRINOX followed by R0 resection in patients with initially unresectable ASCP. However, the patient did not receive a preoperative evaluation for distant metastases such as chest computed tomography (CT) or FDG-PET because of the patient’s financial circumstances, resulting in inaccurate preoperative staging. Adjuvant chemotherapy with gemcitabine and paclitaxel was administered after surgery. Nevertheless, a scheduled abdominal CT scan six months after surgery revealed multiple metastatic lesions in the liver, and the patient died after a few months [31]. Consequently, the case presented here is the first report of successful R0 resection of initially unresectable ASCP after conversion-intended chemotherapy using gemcitabine and nab-paclitaxel. The patient has subsequently remained relapse-free for seven months.

Regarding operative curability in pancreatic cancer surgery, the importance of R0 resection has been confirmed in a randomized phase III trial of PDAC [32]. Versteijne et al. reported that, in 248 patients with resectable or borderline-resectable PDAC who underwent neoadjuvant chemoradiation or immediate surgery, R0 resection was associated with improved overall survival compared with non-R0 resection (HR, 0.47; 95% CI 0.31 to 0.72; *P* < 0.001) [32]. The effect on overall survival of R0 resection after downstaging therapy for unresectable PDAC has been reported by Gillen et al. who performed a meta-analysis of 111 studies that included 4394 patients with PDAC [33]. In that study, of the 147 patients initially staged as unresectable, 33% could undergo successful resection after chemo- and/or radiation therapy with comparable R0 resection rates as that in the group of patients with initially resectable tumors (79% vs. 82%, respectively) and their survival (20.5 months) was comparable with those with initially resectable tumors. The importance of R0 resection of ASCP has also been reported, although in a small number of studies. In a series of 23 patients with ASCP reported by Smoot et al., the completeness of resection affected median survival, which was 14.4 months for R0 resection compared with 8 months for R1 and 4.8 months for palliative care patients who only received medical therapy [27]. However, the effect on prognosis after R0 resection with conversion-intended chemotherapy for unresectable ASCP is currently unclear.

ASCP is rare and difficult to diagnose by radiological imaging studies and may only be finally diagnosed by postoperative pathology, as in the present case. Therefore, for unresectable pancreatic cancer in general, conversion-intended therapy should be considered, and the resectability should be reevaluated after the therapy [2, 25, 33]. However, a histological confirmation achieved through EUS-FNA can facilitate the selection of an optimal treatment plan [29]. If the diagnosis of adenosquamous carcinoma had been made preoperatively in the present case, preoperative radiation therapy might have been included as an effective treatment for the squamous cell carcinoma component [4]. However, in this case, chemotherapy alone was effective and R0 resection was possible, although chemoradiotherapy may be more effective in more locally advanced cases. EUS-FNA would be better to perform whenever possible after considering the risks and benefits [29]. In the present case, the adenocarcinoma component was predominant, with 70% adenocarcinoma and 30% squamous cell carcinoma. Gemcitabine and nab-paclitaxel have been considered to be effective for treating the adenocarcinoma component of ASCP [5]. However, Voong et al. reported that adjuvant chemoradiotherapy for ASCP statistically improved survival in patients with increased squamous component (≥ 30%) [18]. Further investigation is needed regarding the choice of preoperative treatment according to the predominance of tumor components in ASCP. Staging laparoscopy was not performed in this case. However, most patients with radiographically defined locally advanced PDAC have occult distant organ metastasis [34]. Therefore, staging laparoscopy including lavage cytology may be considered in conversion-intended chemotherapy in ASCP in cases where microhepatic metastases or peritoneal dissemination cannot be denied, to provide accurate staging and avoid unnecessary laparotomy [34, 35].

The prognostic impact must be clarified after R0 resection with conversion-intended therapy for unresectable ASCP as well as to establish an effective perioperative therapy for ASCP in the future.

## Conclusions

Conversion-intended chemotherapy with gemcitabine and nab-paclitaxel chemotherapy may be effective for ASCP.

## Data Availability

Not applicable.

## Abbreviations

ASCP: Adenosquamous carcinoma of the pancreas
PDAC: Pancreatic ductal adenocarcinoma
MDCT: Multidetector row computed tomography
FDG-PET: Fluorodeoxyglucose-positron emission tomography
EUS-FNA: Endoscopic ultrasonography-fine needle aspiration
FOLFIRINOX: Leucovorin, 5-fluorouracil, irinotecan, and oxaliplatin
CT: Computed tomography
